# Reliability and validity of the Chinese version of the achievement emotions questionnaire for physical education in university students

**DOI:** 10.1186/s12889-023-16759-5

**Published:** 2023-09-21

**Authors:** Jianing Tian, Peifeng Liu, Qianqian Zhang, Shun Song, Shicheng An, Hongyan Yu

**Affiliations:** 1 Department of Physical Education, Shanghai Jiao Tong University, Shanghai, 200240 China; 2 Department of Physical Education, Central South University, Changsha, 410083 China

**Keywords:** Achievement emotions, Physical education, University students, Reliability, Validity, Measurement invariance

## Abstract

**Background:**

Achievement emotions have a significant impact on both the learning process and outcomes. However, there is currently no brief and effective questionnaire available to evaluate Chinese university students' achievement emotions in physical education courses. This study aimed to examine the reliability and validity of the Achievement Emotions for Physical Education Questionnaire (AEQ-PE) in a sample of Chinese university students, while also investigating its measurement invariance across gender and grade levels.

**Methods:**

A cluster randomization sampling method was used to select 694 first- and second-year university students in Shanghai, China for the survey. Descriptive statistics, item analysis, reliability testing, and measurement invariance testing were conducted on the full sample (*n* = 694). Subsequently, the full sample was randomly divided into two groups, with Sample 1 (*n* = 347) undergoing exploratory factor analysis (EFA), and Sample 2 (*n* = 347) undergoing confirmatory factor analysis (CFA) to test the structural validity, convergent validity, and discriminant validity of the Chinese version of the Achievement Emotions Questionnaire for Physical Education (AEQ-PE-C). Finally, Sample 3 (*n* = 45), which was retested one month later, was used to evaluate test–retest reliability.

**Results:**

The Chinese version of the Achievement Emotions Questionnaire for Physical Education consists of 6 dimensions and 24 items, with good item discrimination. The EFA supported a 6-factor structure model, while the CFA demonstrated good model fit indices (χ2/df = 3.086, CFI = 0.928, TLI = 0.916, RMSEA = 0.078) and good convergent and discriminant validity. The questionnaire exhibits high internal consistency reliability (0.794) and excellent test–retest reliability (0.792). Furthermore, the multi-group analysis confirms that the AEQ-PE-C questionnaire has measurement invariance across gender and grade levels.

**Conclusion:**

The Chinese version of the Achievement Emotions Questionnaire for Physical Education has good reliability and validity, as well as measurement invariance across gender and grade levels, making it an effective tool for measuring achievement emotions in physical education among Chinese university students.

**Supplementary Information:**

The online version contains supplementary material available at 10.1186/s12889-023-16759-5.

## Introduction

Emotions are widely recognized as a crucial factor influencing individuals' behavior and are significantly associated with complex psychological processes such as cognition, motivation, and attitude [1, 2]. The term emotion is defined as an organism's attitudinal experience that reflects the relationship between objective things and the subject's needs [3]. In 1998, the American Educational Research Association held a workshop on "The Role of Emotion in Student Learning and Achievement," which set the stage for subsequent research on the use of emotion in pedagogy. Since then, researchers have increasingly focused on students' achievement emotions [4].

The concept of achievement emotions was first defined in 2002 by the German psychologist Pekrun et al., who stated that achievement emotions is emotions directly linked to school learning, classroom instruction, and academic achievement [5]. Achievement emotions is typically intense and can cause significant effects on students' attention, motivation, use of learning strategies, self-regulation of learning, and academic performance [6–8]. In addition, Pekrun et al. proposed the Control-Value Theory of Achievement Emotions (CVTAE), which extends the categories of achievement emotions and provides a comprehensive framework for analyzing the emotions experienced in learning contexts. To gain an accurate understanding of students' achievement emotions, it is essential to use valid and reliable measurement questionnaires [9]. Based on the CVTAE, the Achievement Emotions Questionnaire (AEQ) was designed by Pekrun in 2005 [10]. This questionnaire has been widely discussed and applied in several countries and regions worldwide [11–13].

However, as research deepens, it has been found that achievement emotions manifest differently in various subjects. Research conducted by Goetz et al. indicates a weak and inconsistent relationship among the academic emotions experienced by students in mathematics, physics, German, and English subjects [14]. Therefore, AEQ was developed by later scholars as a tool to measure students' emotions in different subjects, such as the Academic Emotions Questionnaire-Mathematics (AEQ-M) [15] and the Achievement Emotions Questionnaire-Foreign Language Class (AEQ-FLC) [16]. As a unique subject, physical education places a dual changes on students, demanding both mental and physical engagement. Consequently, emotions experienced during physical education learning exhibit greater depth and breadth than those experienced in other subjects. In recent years, there has been a growing body of international research focused specifically on the study of achievement emotions in physical education [17–19].

Currently, regarding the measurement of achievement emotions in physical education, Trigueros et al. have developed The Scale of Emotions in Physical Education (SEPE) through expert survey methods, which includes 8 categories of emotions with a total of 40 items [20]. However, this scale lacks theoretical support and does not provide a clear explanation of the inherent logical relationships between the indicators included in the scale. Simonton et al. have revised the AEQ questionnaire for physical education to create the Discrete Emotions in Physical Education Scale (DEPES), which focuses solely on three categories of negative emotions—boredom, shame, and anger—without accounting for positive emotions [21]. Notably, Fierro-Suero et al. have revised the AEQ questionnaire to form the Achievement Emotions Questionnaire for Physical Education (AEQ-PE), which comprises 24 questions and encompasses six emotions: pride, enjoyment, anger, anxiety, hopelessness, and boredom [22]. Each emotion can be further divided into three dimensions. The first dimension is valence, which categorizes emotions into positive and negative, such as enjoyment as a positive emotion and anxiety as a negative emotion. The second dimension is arousal level, which divides emotions into high and low arousal levels, such as high arousal positive emotions (enjoyment and pride). The third dimension is object focus, which divides emotions into activity emotions and outcome emotions. Outcome emotions include prospective outcome emotions (hope and anxiety) and retrospective outcome emotions (pride). The AEQ-PE has been translated into multiple languages (including Spanish, English, and Malay) and has been shown to have good reliability and validity in several countries [19, 22, 23].

China has a vast number of university students, with the current number of university students exceeding 44.3 million, constituting one-fifth of the total number of university students worldwide [24]. According to the "Basic Standards for Physical Education in Higher Education Institutions" issued by the Chinese Ministry of Education, first- and second-year undergraduate students are required to take at least 144 h of physical education courses, with a minimum of 2 h per week and no less than 45 min per class [25]. Despite such a huge teaching group for physical education courses in Chinese universities, there is currently no brief and effective questionnaire available to evaluate the achievement emotions of university students in physical education courses. So far, the only questionnaire that measures achievement emotions in Chinese physical education classes is the General College Student Physical Education Achievement Emotion Scale revised by Yang based on the AEQ questionnaire, which comprises a total of 56 questions [26]. While this scale provides a comprehensive evaluation of achievement emotions in physical education, its limitations are also apparent. For instance, the large number of questions may result in longer response times, leading to response fatigue and bias, and may also reduce response rates. This may be one of the reasons why the questionnaire has not been widely adopted.

In summary, this study takes the AEQ-PE questionnaire developed by Fierro-Suero as its benchmark. It examines the reliability and validity of Chinese version of the Achievement Emotions Questionnaire for Physical Education (AEQ-PE-C) and explores its measurement invariance across gender and academic years. Based on this, the study proposes the following hypotheses: the revised AEQ-PE-C questionnaire demonstrates strong applicability and effectiveness, serving as a robust tool for in-depth investigation of Chinese university students' academic emotions in physical education. This questionnaire can provide researchers with a preliminary understanding of academic emotions in sports and lay the foundation for subsequent relevant academic research. Through the utilization of this questionnaire, physical education teachers and researchers can gain profound insights into university students' academic emotional states, enabling targeted adjustments of pedagogical strategies based on different emotional characteristics, thereby significantly enhancing the effectiveness of education.

## Methods

### Participants

In China, Physical education classes are mandatory for first- and second-year undergraduate students. Therefore, first- and second-year undergraduate students in Shanghai were selected as the target population for this study. The survey questionnaire was designed using the online platform "Wenjuanxing," which is the functional equivalent of the Amazon Mechanical Turk platform. We employed a cluster randomization sampling method within classes and distributed the questionnaire via a combination of online and offline methods from October to November 2022. The exclusion criteria for the questionnaire included non-freshman and sophomore students, missing data values, and regularity or continuity in responses. A total of 707 questionnaires were collected, of which 13 were invalid and excluded from the analysis. Finally, 694 valid questionnaires were included, with a valid response rate of 98.16%. The study protocol adhered to the guidelines of the Declaration of Helsinki and was approved by the Science and Technology Ethics Committee of Shanghai Jiao Tong University. All participants completed the questionnaires voluntarily.

To meet the requirements of data analysis, the full sample data of subjects (*n* = 694) was randomly divided into two parts: sample 1 (*n* = 347) and sample 2 (*n* = 347). Additionally, 50 subjects from the full sample were randomly selected for a test–retest of the questionnaire after a one-month interval. Following the elimination of invalid questionnaires, 45 matched pairs of data were obtained to form sample 3.

### Instruments

The Achievement Emotions Questionnaire for Physical Education (AEQ-PE) was developed by Fierro-Suero et al. [22]. It comprises 24 items divided into 6 categories of achievement emotions, including enjoyment, pride, anger, anxiety, hopelessness, and boredom, each encompassing 4 items. The questionnaire utilizes a 5-point Likert scale, ranging from 1 (completely disagree) to 5 (completely agree), to score responses. Higher scores in the categories of enjoyment and pride indicate a greater degree of positive emotions, while higher scores in the categories of anger, anxiety, hopelessness, and boredom suggest a higher degree of negative emotions.

### Translation procedure

The AEQ-PE questionnaire was initially translated into Chinese by two graduate students proficient in both Chinese and English, and with a background in sports science. During the translation process, these two students actively sought guidance from professional translators. When necessary, they engaged in consultations and discussions to ensure the accuracy of each translation. Subsequently, a professor of psychology and a professor of physical education were consulted to refine the language presentation and account for cultural differences. To ensure accuracy and consistency, native English-speaking international students were then invited to back-translate the Chinese version into English, and each item was compared with the original English version. This rigorous process determined the accuracy of the AEQ-PE-C presentation, and the final version of the questionnaire was completed.

### Statistical analysis

The data collected were analyzed using statistical analysis software, specifically SPSS 26.0 and AMOS 23.0.

In the full sample (*n* = 694), descriptive statistics were conducted to analyze the participants' demographic characteristics and scores on each questionnaire item. The item analysis first calculates the correlation coefficient between each item and the total score, with a minimum critical value of r ≥ 0.4. Subsequently, using the total score sorting method, the top 27% of scores are categorized as the high-score group, and the lowest 27% as the low-score group. Following this, an independent two-tailed t-test is conducted. If the scores of each item in both groups reach a significant level, it is considered as indicating a good discriminant validity [27]. Cronbach alpha coefficients were calculated to test the questionnaire's internal consistency. Hair et al. (1988) suggested that a Cronbach's alpha coefficient greater than 0.7 indicates high reliability of the questionnaire [28]. Additionally, a series of nested models were constructed in Amos 23.0 to test the measurement invariance of the questionnaire across gender and grade levels.

In sample 1 (*n* = 347), Kaiser–Meyer–Olkin (KMO) values and Bartlett's sphericity test were utilized to assess the suitability of the data for factor analysis. The assessment criteria are a Kaiser–Meyer–Olkin (KMO) value greater than 0.60 and the significant result of the Bartlett's test of sphericity [29, 30]. Exploratory factor analysis (EFA) was then conducted to initially test the factor structure and factor loading of the questionnaire.

In sample 2 (*n* = 347), we conducted Confirmatory Factor Analysis (CFA) to assess the construct validity of the questionnaire. The criteria for evaluating the fit of construct models are as follows: the chi-square degree of freedom (χ^2^/df) should be less than 5, indicating reasonable fit; the Comparative Fit Index (CFI) and Tucker Lewis Index (TLI) should both exceed 0.9, signifying good fit; the Standardized Root Mean Square Residual (SRMR) is considered good if values are below 0.08; and the Root Mean Square Error of Approximation (RMSEA) should not exceed 0.08 for well-fitted models [31–33]. Convergent validity of the model was assessed through two measures, namely Average Variance Extracted (AVE) and Construct Reliability (CR). According to Fornell and Larcker (1981), AVE > 0.50 and CR ≥ 0.70 are considered acceptable [34]. To assess the discriminant validity of the model, the square root of the Average Variance Extracted (AVE) was used. If the square root of the AVE of a factor is greater than the correlation coefficient of that factor with all other factors, it indicates good discriminant validity [35, 36].

In sample 3 (*n* = 45), we utilized the intra-class correlation coefficient (ICC) to evaluate the test–retest reliability of the questionnaire. An ICC above 0.7 is generally considered to indicate good test–retest reliability of the questionnaire [37]. This analysis aimed to determine the stability and consistency of scores obtained from the questionnaire across various time points.

## Results

### Descriptive statistics

Table 1 provides an overview of the demographic characteristics of the study participants, including gender, Ethnical groups, registered residence, and grade. Meanwhile, Table 2 presents the Mean (SD) scores for each subdomains and items of the AEQ-PE-C questionnaire.

**Table 1:** Demographic characteristics of the participants (*n* = 694)

Characteristic (*n* = 694)	n	%
**Gender**		
Male	477	68.7
Female	217	31.3
**Ethnical groups**		
Han nationality	533	76.8
Ethic minority	161	23.2
**Registered residence**		
Urban	558	80.4
Rural	136	19.6
**Grade**		
Freshman	498	71.8
Sophomore	196	28.2

**Table 2:** Mean (SD) scores for each subdomains and items of the AEQ-PE-C (*n* = 694)

Subdomains and Items	Mean (SD)
Pride	4.34 (0.77)
1. I am proud to be able to keep up with the physical education class	4.37 (0.87)
2. I am proud of my participation in a physical education class	4.39 (0.85)
3. I think that I can be proud of what I know about physical education	4.26 (0.93)
4. Because I take pride in my accomplishments in physical education, I am motivated to continue	4.34 (0.87)
Enjoyment	4.28 (0.77)
5. I am motivated to go to the physical education class because it is exciting	4.06 (1.01)
6. I enjoy being in the physical education class	4.29 (0.89)
7. I feel excited about being in physical education class, practicing what the teacher suggests	4.31 (0.84)
8. I am glad going to the physical education class paid off	4.48 (0.75)
Anger	1.53 (0.74)
9. I feel anger welling up in me during the physical education class	1.70 (0.98)
10. Because I am angry, I get restless in the physical education class	1.50 (0.81)
11. Thinking about all the useless things I have to learn in physical education, annoys me	1.57 (0.87)
12. After the physical education class, I am angry	1.35 (0.72)
Anxiety	2.07 (0.93)
13. I worry that the things I have to do in physical education classes might be too difficult	2.37 (1.25)
14. I feel nervous in the physical education class	2.02 (1.11)
15. I get scared that I might say/do something wrong in the physical education class, and I would rather not say/do anything	1.73 (0.95)
16. When I do not understand something in the physical education class, my heart races	2.15 (1.14)
Hopelessness	1.48 (0.68)
17. It is pointless to prepare for the physical education class because I am bad at it anyway	1.59 (0.82)
18. Even before entering the physical education class, I know I will not get it right	1.51 (0.79)
19. I would rather not go to the physical education class because it is impossible to perform the exercises correctly	1.44 (0.75)
20. I have lost all hope of doing physical education activities effectively	1.37 (0.74)
Boredom	1.43 (0.70)
21. I feel like leaving during the physical education class because it is so boring	1.45 (0.76)
22. I get bored during the physical education class	1.47 (0.78)
23. The physical education class bores me	1.42 (0.75)
24. I find the physical education class fairly dull	1.40 (0.69)

### Item analysis

After reversing the scores for the items related to boredom, hopelessness, anxiety, and anger, an item-to-total score correlation analysis was conducted. The findings indicated a significant correlation between each item and the total score, with correlation coefficients ranging from 0.54 to 0.79 (*p* < 0.001). Furthermore, independent sample t-tests were conducted for the high and low groups, revealing significant differences in scores for all items (*p* < 0.001). These results suggest that the AEQ-PE-C items were well-discriminated, as illustrated in Table 3.

**Table 3:** Results of item analysis (*n* = 694)

Items	*r*	*t*	Items	*r*	*t*
1	0.67^***^	18.02^***^	13	0.65^***^	25.15^***^
2	0.67^***^	18.56^***^	14	0.71^***^	24.28^***^
3	0.60^***^	16.58^***^	15	0.72^***^	22.09^***^
4	0.67^***^	18.77^***^	16	0.54^***^	16.86^***^
5	0.65^***^	22.06^***^	17	0.75^***^	22.39^***^
6	0.73^***^	20.21^***^	18	0.77^***^	20.23^***^
7	0.66^***^	20.65^***^	19	0.79^***^	19.18^***^
8	0.67^***^	18.11^***^	20	0.75^***^	15.60^***^
9	0.62^***^	15.67^***^	21	0.77^***^	18.21^***^
10	0.70^***^	18.16^***^	22	0.76^***^	17.43^***^
11	0.73^***^	19.91^***^	23	0.79^***^	17.95^***^
12	0.73^***^	14.75^***^	24	0.77^***^	17.14^***^

^***^ *p* < 0.001

### Exploratory factor analysis

To examine the factor structure and factor loading of the data from sample 1 (*n* = 347), exploratory factor analysis (EFA) was conducted. The results in Table 4 indicate that the Kaiser–Meyer–Olkin (KMO) value was 0.945, and the Bartlett's sphericity test was significant (χ2 = 6326.614, df = 276, *p* < 0.001). These findings demonstrate that the data meet the necessary requirements for factor analysis, as the KMO value exceeds the recommended criterion of 0.6, and the Bartlett's sphericity test is significant.

**Table 4:** KMO values and Bartlett's spherical test of the questionnaire (*n* = 347)

KMO values and Bartlett's spherical test		
KMO		0.952
	χ2	6326.614
Bartlett's spherical test	*df*	276
	*p*	0.000

Given that the AEQ-PE-C measures six dimensions of achievement emotions, we initially extracted six common factors to ensure consistency between the Chinese version of the questionnaire and the original version. Principal component analysis was then performed, revealing that the cumulative variance explained by the six common factors with eigenvalues greater than 1 was 79.157% (see Appendix 1). Subsequently, the maximum variance method was used to orthogonal rotate the factors, and the overall factor loadings of the questionnaire ranged from 0.532 to 0.848. Specific factor loadings for each dimension were as follows: pride (dimension 1) = 0.805–0.846; enjoyment (dimension 2) = 0.699–0.797; anger (dimension 3) = 0.650–0.812; anxiety (dimension 4) = 0.613–0.803; hopelessness (dimension 5) = 0.532–0.684; boredom (dimension 6) = 0.758–0.848 (see Appendix 2 for detailed results). It is important to note that the EFA output differed from the expected factor structure, as the two dimensions of pride and enjoyment, constructed by the original questionnaire, converged into one dimension. However, given the definition of both emotions, the two dimensions are somewhat distinct. Therefore, we conducted EFA again for both emotions by extracting two fixed factors. The results showed that the four items related to pride (factor loadings:0.682–0.854) and the four items related to enjoyment (factor loadings:0.722–0.853) loaded onto the two dimensions respectively, which was consistent with the expected structure (see Appendix 3). Thus, we chose to retain the original 6-factor model.

### Confirmatory factor analysis

**Fig. 1:**
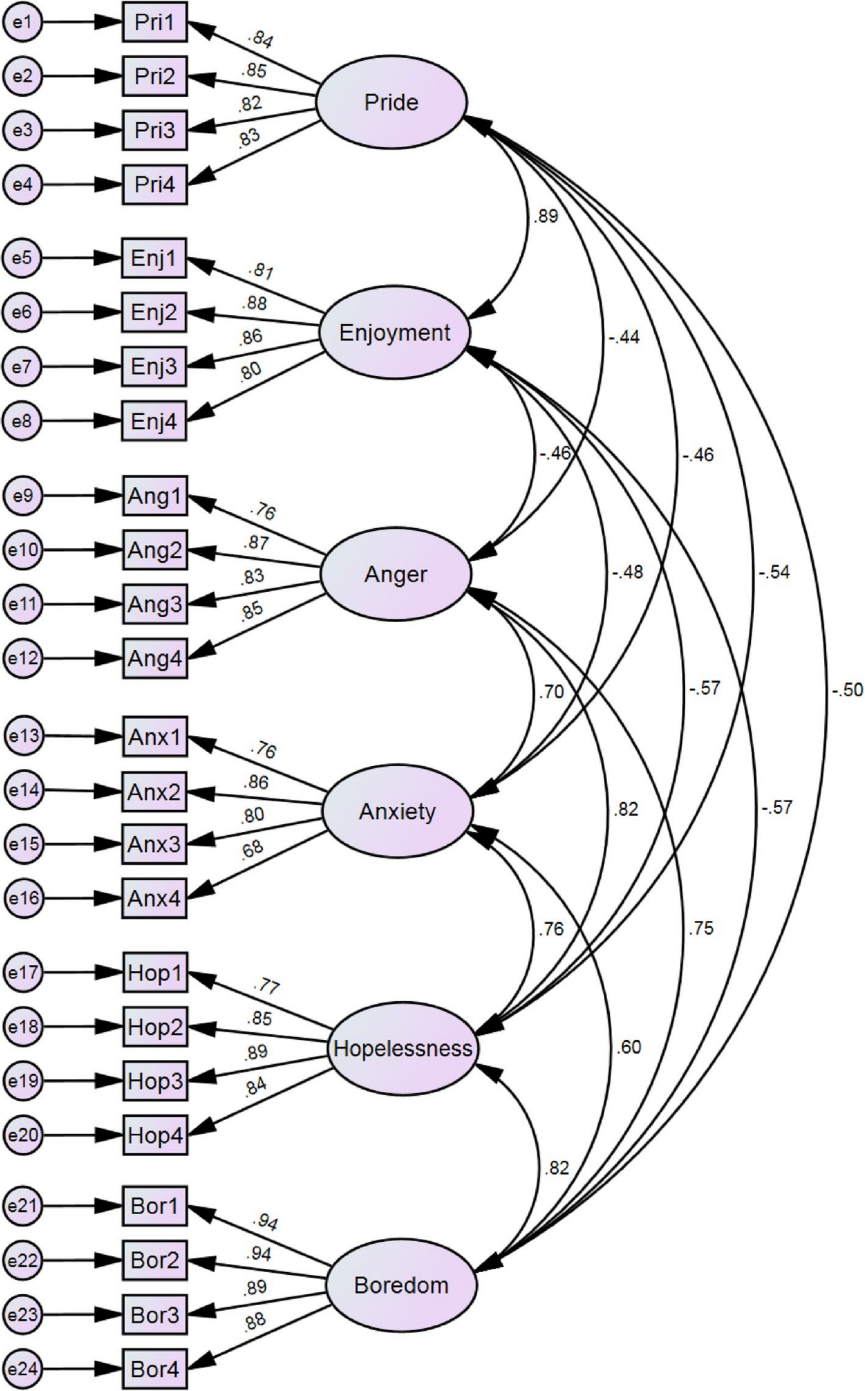
First-order six-factor structural model

In order to test the validity of the AEQ-PE-C (Fig. 1), a first-order six-factor confirmatory factor analysis model was constructed using the data from sample 2 (*n* = 347). The fit indices of the model were analyzed and found to meet the statistical criteria, indicating that the model was a good fit and demonstrated good construct validity for the AEQ-PE-C. Please refer to Table 5 for further details.

**Table 5:** Fitting indicators of the first-order six-factor structural model (*n* = 347)

Model	χ2	df	χ2/df	CFI	TLI	SRMR	RMSEA (90% CI)
6-factor model	731.301	237	3.086	0.928	0.916	0.053	0.078 (0.071–0.084)
Fitness criteria			< 5	≥ 0.90	≥ 0.90	< 0.08	< 0.08

χ2 chi-square, *df* degree of freedom, *CFI* Comparative Fit Index, *TLI* Tucker Lewis Index, *SRMR* Standardized Root Mean Square Residual, *RMSEA* Root Mean Square Error of Approximation, *90% CI* 90% Confidence Interval

### Convergent and discriminant validity

As shown in Table 6, the factor loadings of the items corresponding to the six factors of pride, enjoyment, anger, anxiety, hopelessness, and boredom exceeded 0.6, indicating high representativeness of the items. Furthermore, all six dimensions had AVE values (ranging from 0.606 to 0.834) greater than 0.5 and CR values (ranging from 0.859 to 0.953) greater than 0.7, indicating ideal convergent validity of the AEQ-PE-C.

**Table 6:** Convergent validity of the AEQ-PE-C model (*n* = 347)

Path			Estimate	AVE	CR
Pri4	< –-	Pride	0.826	0.695	0.901
Pri3	< –-	Pride	0.821		
Pri2	< –-	Pride	0.849		
Pri1	< –-	Pride	0.838		
Enj4	< –-	Enjoyment	0.799	0.701	0.903
Enj3	< –-	Enjoyment	0.859		
Enj2	< –-	Enjoyment	0.882		
Enj1	< –-	Enjoyment	0.806		
Ang4	< –-	Anger	0.847	0.684	0.896
Ang3	< –-	Anger	0.833		
Ang2	< –-	Anger	0.865		
Ang1	< –-	Anger	0.759		
Anx4	< –-	Anxiety	0.683	0.606	0.859
Anx3	< –-	Anxiety	0.796		
Anx2	< –-	Anxiety	0.864		
Anx1	< –-	Anxiety	0.759		
Hop4	< –-	Hopelessness	0.844	0.706	0.906
Hop3	< –-	Hopelessness	0.890		
Hop2	< –-	Hopelessness	0.848		
Hop1	< –-	Hopelessness	0.775		
Bor4	< –-	Boredom	0.882	0.834	0.953
Bor3	< –-	Boredom	0.895		
Bor2	< –-	Boredom	0.937		
Bor1	< –-	Boredom	0.938		

*AVE* Average Variance Extracted, *CR* Construct Reliability

As shown in Table 7, significant correlations were found among the six dimensions of pride, enjoyment, anger, anxiety, hopelessness, and boredom (*p* < 0.001). Furthermore, the absolute values of the correlation coefficients were all less than 0.5 and were all less than the corresponding square root of AVE. These results demonstrate a good degree of discrimination among the dimensions, indicating that the AEQ-PE-C has ideal discriminant validity.

**Table 7:** Discriminant validity of the AEQ-PE-C model (*n* = 347)

	1	2	3	4	5	6
1.Pride	0.695					
2.Enjoyment	0.378^***^	0.701				
3.Anger	0.190^***^	0.167^***^	0.684			
4.Anxiety	0.255^***^	0.223^***^	0.332^***^	0.606		
5.Hopelessness	0.238^***^	0.211^***^	0.306^***^	0.367^***^	0.706	
6.Boredom	0.217^***^	0.208^***^	0.278^***^	0.287^***^	0.310^***^	0.834
AVE square root	0.834	0.837	0.827	0.778	0.840	0.913

^***^ *p* < 0.001

### Reliability analysis

To evaluate the reliability of the AEQ-PE-C, two indicators were used: internal consistency reliability (Cronbach's alpha) and test–retest reliability (intra-class correlation coefficient, ICC). The Cronbach's alpha coefficients for the six dimensions of the AEQ-PE-C ranged from 0.853 to 0.952, with an overall Cronbach's alpha coefficient of 0.794. Furthermore, the reliability of each individual item ranges from 0.770 to 0.811, as detailed in Appendix 4. These results demonstrate good internal consistency reliability of the AEQ-PE-C. Additionally, test-retesting 45 subjects one month after the initial test revealed an ICC of 0.792 for the total questionnaire and between 0.690 and 0.828 for each of the six dimensions. These results suggest that the AEQ-PE-C has good test–retest reliability. Please refer to Table 8 for further details.

**Table 8:** Internal consistency reliability and test–retest reliability of AEQ-PE-C

	Total Score	Factor 1	Factor 2	Factor 3	Factor 4	Factor 5	Factor 6
Cronbach α	0.794	0.901	0.898	0.889	0.853	0.902	0.952
ICC	0.792	0.690	0.818	0.808	0.828	0.741	0.810

*ICC* intra-class correlation coefficient

### Measurement invariance testing

The test followed four progressively more stringent steps (configural invariance, metric invariance, scalar invariance, and strict invariance), as outlined in Table 9. Measurement invariance was determined based on the amount of variation in Comparative Fit Index (CFI) and Root Mean Square Error of Approximation (RMSEA) between models. Specifically, a difference of less than 0.01 in both CFI and RMSEA (|ΔCFI|< 0.01 and |ΔRMSEA|< 0.01) indicates invariance [38].

**Table 9:** AEQ-PE-C measurement invariance fit indices and model comparison results (*n* = 694)

Model	Fit Indices					Model Comparison		
	χ2	*df*	TLI	CFI	RMSEA	Δχ2 (*df*)	|ΔCFI|	|ΔRMSEA|
Gender								
Model 1	1553.396	474	0.915	0.927	0.057			
Model 2	1572.498	492	0.918	0.927	0.056	19.10 (18)^a^	0	0.001
Model 3	1677.582	513	0.915	0.921	0.057	124.19 (39)^b^	0.006	0.001
Model 4	1803.95	537	0.912	0.914	0.058	250.56 (63)^c^	0.007	0.001
Grade								
Model 1	1637.352	474	0.908	0.921	0.06			
Model 2	1666.627	492	0.911	0.92	0.059	29.28 (18)^a^	0.001	0.001
Model 3	1784.099	513	0.907	0.914	0.06	117.47 (21)^b^	0.006	0.001
Model 4	1862.118	537	0.908	0.91	0.06	78.019 (24)^c^	0	0

*Model 1* Configural Invariance, *Model 2* Metric invariance, *Model 3* Scalar invariance, *Model 4* Strict Invariance

^a^ indicates Model 2 vs Model 1

^b^ indicates Model 3 vs Model 2

^c^ indicates Model 4 vs Model 3

The cross-gender invariance test was conducted in four steps. First, a structural equivalence model (configural invariance) was established, and the fit indices of this model reached an acceptable level, satisfying the conditions for conducting the invariance test. Second, a factor loading equivalence model (metric invariance) was established, and the difference values between model 2 and model 1 (ΔCFI = 0 and ΔRMSEA = 0.001) were less than the standard values, confirming the factor loading cross-gender equivalence. Third, an intercept equivalence model (scalar invariance) was constructed and compared with model 2, with the difference values (ΔCFI = 0.006 and ΔRMSEA = 0.001) smaller than the standard values, indicating that the assumption of intercept cross-gender equivalence was valid. Finally, a residual equivalence model (strict invariance) was constructed and compared with model 3, and the difference values (ΔCFI = 0.007 and ΔRMSEA = 0.001) were both smaller than the standard value, indicating that the residual cross-gender equivalence holds. These results suggest that the AEQ-PE-C six-factor model has measurement invariance between groups of different genders.

Similarly, in the cross-grade invariance test, we established four models to evaluate the measurement invariance of the AEQ-PE-C six-factor model across different grade groups. The first model, the structural equivalence model 1, showed good fit indices and met the requirements for the invariance test. The second model, the factor loading equivalence model 2, confirmed that the cross-grade equivalence of factor loadings held as the difference values between model 2 and model 1 were smaller than the standard values (ΔCFI = 0.001 and ΔRMSEA = 0.001). The third model, the intercept equivalence model 3, demonstrated that the assumption of intercept equivalence across grades was valid, as the difference values were smaller than the standard value (ΔCFI = 0.006 and ΔRMSEA = 0.001). Finally, the fourth model, the residual equivalence model 4, showed that the residual cross-grade equivalence held as the difference values were smaller than the standard value (ΔCFI = 0.004 and ΔRMSEA = 0). These results indicate that the AEQ-PE-C six-factor model has measurement invariance across grade groups.

## Discussion

This study aimed to translate and revise the AEQ-PE questionnaire, resulting in the development of a brief Chinese version of the Achievement Emotions Questionnaire for Physical Education (AEQ-PE-C) suitable for Chinese university students. Furthermore, we conducted reliability and validity tests on the AEQ-PE-C questionnaire and assessed its measurement invariance across gender and grade groups.

The results of the study indicated that the AEQ-PE-C had good item discrimination. The overall questionnaire, its six dimensions, and individual items all exhibit good performance in terms of internal consistency, indicating strong reliability. Additionally, the questionnaire also demonstrated good test–retest reliability over a one-month interval, indicating its good temporal stability. The revised AEQ-PE-C questionnaire consisted of 24 items across six dimensions, including pride, enjoyment, anger, anxiety, boredom, and hopelessness. The dimensions and corresponding items of the AEQ-PE-C were consistent with the original scale. However, it should be noted that during exploratory factor analysis, the pride and enjoyment dimensions were grouped into the same factor. Although both pride and enjoyment are positive emotions, based on the control-value theory upon which the original scale was constructed, pride is considered a retrospective outcome emotion, while enjoyment is defined as an activity emotion with a different object focus [39]. Simultaneously, within China's social values and emotional expression, enjoyment is often closely associated with positive emotional experiences, inner joy, and a liking for the activity itself. In contrast, pride in Chinese culture places a stronger emphasis on an individual's sense of achievement, performance, or behavior, giving rise to feelings of pride and self-assurance. Given these distinctions, we employed the expert consultation method, seeking experts' opinions on separating these two dimensions, which received validation. Therefore, we subsequently conducted exploratory factor analysis on the enjoyment (four items) and pride (four items) dimensions, and the results showed that each item loaded onto its corresponding dimension. Consequently, we continued to use the six-factor structure in subsequent analyses. Given that EFA is primarily employed for preliminary data analysis, subsequently, we conducted CFA to revalidate the model's factor structure. Concurrently, we assessed convergent validity by calculating AVE values and CR values, while also examining discriminant validity using the square root of AVE. The research findings indicate that the questionnaire demonstrates a favorable level of structural validity. As a result, the AEQ-PE-C can be considered an effective tool for assessing the achievement emotions of physical education among Chinese university students.

The recent revision of the Malaysian version of the AEQ-PE questionnaire mentioned, among the limitations, that future revisions of this questionnaire should consider invariance testing to provide further evidence for the questionnaire's validity [23]. As such, another significant contribution of this study was to investigate whether the AEQ-PE-C maintained the same meaning and underlying structure across various groups, including gender and grade level. The study results indicate that the AEQ-PE-C exhibits gender measurement invariance, which was established after conducting a series of tests. Firstly, the model constructed separately with data from male and female participants demonstrated equivalent structural relationships between the six dimensions of the AEQ-PE-C and their corresponding items. This is a prerequisite for meaningful subsequent steps in the measurement invariance test. Secondly, the relationship between the measurement items and the dimensions of the AEQ-PE-C is equivalent across gender groups, indicating that males and females have a consistent understanding of the construct. Thirdly, the intercept between observed variables is invariant, which implies that males and females have the same systematic tendency to understand the content of the measure. Fourthly, the residual equivalence of individual terms between the two groups suggests that the actual scores of the test variables are consistent between males and females. Additionally, cross-grade measurement invariance for the AEQ-PE-C was also demonstrated across different grades. Therefore, the revised questionnaire in this study can be used to examine the characteristics of achievement emotions in physical education among Chinese university students across genders and grade levels.

Compared to the revised "General College Student Physical Education Achievement Emotion Scale" by Li Yangyang, the AEQ-PE-C questionnaire used in this study has fewer items, making it more convenient to use and saving time and effort. Additionally, following the validation and reliability testing, this questionnaire underwent tests for measurement invariance across different grade levels and genders, as well as a test for temporal stability. These characteristics provide a brief and effective measurement tool for assessing academic emotions in physical education instruction.

Currently, numerous international studies have demonstrated that achievement emotions in physical education play a crucial role in the curriculum and learning experience [40–42]. According to a study, pride, enjoyment, and hopelessness are the primary emotions that explain physical activity intentions, while enjoyment and boredom significantly impact academic performance [19]. Another study has highlighted the significant correlation between students' achievement emotions in physical education and their classroom participation [43]. Therefore, the revised AEQ-PE-C questionnaire holds educational significance for physical education instructors and researchers in various aspects. It allows researchers to gain a preliminary understanding of the domain of academic emotions in physical education, providing a foundation for future explorations into influencing factors, mechanisms, and related aspects. The questionnaire can accurately capture each student's emotional states and needs, offering personalized guidance and support. Moreover, it enables insight into students' emotional requirements during physical education classes, assisting them in better recognizing, understanding, and managing negative emotions. This ultimately enhances their mental well-being and enriches their experiences in physical activities. Additionally, the questionnaire allows for the comprehension of changes in student emotions. It assists instructors in optimizing course design and teaching methods, ultimately contributing to the creation of a positive and efficient learning environment.

There are several limitations to this study. Firstly, the survey population is limited to university students in Shanghai, China, and may not fully represent the situation of Chinese university students as a whole. Future studies should aim to expand the sample to include university students from other regions in China. Secondly, the reliability analysis of the AEQ-PE-C questionnaire was based on a small sample size of only 45 subjects who participated in the test–retest. As a result, the reliability of the study results may be compromised. Future studies should consider increasing the sample size of subjects to improve the reliability of the study results. Thirdly, this study employed a cross-sectional research design. In the future, a longitudinal approach could be adopted to accurately explore individual emotions related to physical education and their evolving processes, thereby offering more targeted and practical guidance for physical education instructors. Fourthly, the sample of this study exhibited a relatively low representation of females, ethnic minorities, individuals from rural backgrounds, and sophomore students. This could potentially undermine the extent to which these findings can be applied to these specific demographic groups. Hence, it is recommended that future research endeavors incorporate additional validation measures.

## Conclusion

The Chinese version of the Achievement Emotions Questionnaire for Physical Education (AEQ-PE-C) consists of 6 dimensions and 24 items. The questionnaire has demonstrated good reliability and validity and exhibits measurement invariance across both gender and grade groups, making it an effective tool for measuring achievement emotions related to physical education among Chinese university students.

### Supplementary Information

**Additional file 1:** **Appendix 1.** Principal component analysis. **Appendix 2.** Component matrix after orthogonal rotation (24 items). **Appendix 3.** Component matrix after orthogonal rotation (8 items). **Appendix 4.** Reliability of individual items.

## Data Availability

The datasets used and/or analyzed during the current study available from the corresponding author on reasonable request.
