# Supplementary material for: Reliability and validity of the Chinese version of the achievement emotions questionnaire for physical education in university students
Source: BMC Public Health. 2023 Sep 21;23:1839. doi: 10.1186/s12889-023-16759-5 (PMC10512530; doi:10.1186/s12889-023-16759-5)
Supplement: Supplementary file 1 — Additional file 1: Appendix 1. Principal component analysis. Appendix 2. Component matrix after orthogonal rotation (24 items). Appendix 3. Component matrix after orthogonal rotation (8 items). Appendix 4. Reliability of individual items. [file 12889_2023_16759_MOESM1_ESM.docx]

**Appendix 1：Principal component analysis**

| Factor | Initial eigenvalues |  | Sum of squared loadings after extraction | | | Sum of squared loadings after rotation | | |  |
| --- | --- | --- | --- | --- | --- | --- | --- | --- | --- |
|  | Total | Variance percentage | Cumulative% | Total | Variance percentage | Cumulative % | Total | Variance percentage | Cumulative% |
| 1 | 12.035 | 50.145 | 50.145 | 12.035 | 50.145 | 50.145 | 5.530 | 23.041 | 23.041 |
| 2 | 3.134 | 13.059 | 63.204 | 3.134 | 13.059 | 63.204 | 3.992 | 16.634 | 39.676 |
| 3 | 1.492 | 6.217 | 69.421 | 1.492 | 6.217 | 69.421 | 3.240 | 13.5 | 53.176 |
| 4 | 1.013 | 4.220 | 73.641 | 1.013 | 4.22 | 73.641 | 3.066 | 12.777 | 65.952 |
| 5 | 0.705 | 2.936 | 76.577 | 0.705 | 2.936 | 76.577 | 2.354 | 9.809 | 75.762 |
| 6 | 0.619 | 2.580 | 79.157 | 0.619 | 2.58 | 79.157 | 0.815 | 3.395 | 79.157 |
| 7 | 0.529 | 2.206 | 81.363 |  |  |  |  |  |  |
| 8 | 0.442 | 1.842 | 83.205 |  |  |  |  |  |  |
| 9 | 0.400 | 1.669 | 84.874 |  |  |  |  |  |  |
| 10 | 0.388 | 1.618 | 86.492 |  |  |  |  |  |  |
| 11 | 0.354 | 1.477 | 87.969 |  |  |  |  |  |  |
| 12 | 0.318 | 1.327 | 89.296 |  |  |  |  |  |  |
| 13 | 0.299 | 1.244 | 90.540 |  |  |  |  |  |  |
| 14 | 0.285 | 1.188 | 91.728 |  |  |  |  |  |  |
| 15 | 0.283 | 1.179 | 92.907 |  |  |  |  |  |  |
| 16 | 0.263 | 1.094 | 94.001 |  |  |  |  |  |  |
| 17 | 0.239 | 0.995 | 94.996 |  |  |  |  |  |  |
| 18 | 0.222 | 0.926 | 95.921 |  |  |  |  |  |  |
| 19 | 0.218 | 0.908 | 96.829 |  |  |  |  |  |  |
| 20 | 0.184 | 0.767 | 97.596 |  |  |  |  |  |  |
| 21 | 0.175 | 0.729 | 98.325 |  |  |  |  |  |  |
| 22 | 0.173 | 0.722 | 99.047 |  |  |  |  |  |  |
| 23 | 0.143 | 0.594 | 99.641 |  |  |  |  |  |  |
| 24 | 0.086 | 0.359 | 100 |  |  |  |  |  |  |

**Appendix 2：Component matrix after orthogonal rotation (24 items)**

|  | 1 | 2 | 3 | 4 | 5 | 6 |
| --- | --- | --- | --- | --- | --- | --- |
| Pri1 | 0.821 |  |  |  |  |  |
| Pri2 | 0.837 |  |  |  |  |  |
| Pri3 | 0.846 |  |  |  |  |  |
| Pri4 | 0.805 |  |  |  |  |  |
| Enj1 | 0.699 |  |  |  |  |  |
| Enj2 | 0.715 |  |  |  |  |  |
| Enj3 | 0.797 |  |  |  |  |  |
| Enj4 | 0.719 |  |  |  |  |  |
| Ang1 |  | 0.805 |  |  |  |  |
| Ang2 |  | 0.812 |  |  |  |  |
| Ang3 |  | 0.685 |  |  |  |  |
| Ang4 |  | 0.65 |  |  |  |  |
| Anx1 |  |  | 0.761 |  |  |  |
| Anx2 |  |  | 0.779 |  |  |  |
| Anx3 |  |  | 0.613 |  |  |  |
| Anx4 |  |  | 0.803 |  |  |  |
| Hop1 |  |  |  | 0.532 |  |  |
| Hop2 |  |  |  | 0.683 |  |  |
| Hop3 |  |  |  | 0.663 |  |  |
| Hop4 |  |  |  | 0.684 |  |  |
| Bor1 |  |  |  |  | 0.828 |  |
| Bor2 |  |  |  |  | 0.848 |  |
| Bor3 |  |  |  |  | 0.758 |  |
| Bor4 |  |  |  |  | 0.780 |  |

*Note*: the rotation has converged after 6 iterations.

**Appendix 3：Component matrix after orthogonal rotation (8 items)**

|  | 1 | 2 |
| --- | --- | --- |
| Pri1 |  | 0.854 |
| Pri2 |  | 0.758 |
| Pri3 |  | 0.803 |
| Pri4 |  | 0.682 |
| Enj1 | 0.811 |  |
| Enj2 | 0.853 |  |
| Enj3 | 0.748 |  |
| Enj4 | 0.722 |  |

*Note*: the rotation has converged after 6 iterations.

**Appendix 4：Reliability of individual items**

| Items | Cronbach's Alpha |
| --- | --- |
| Pri1 | 0.808 |
| Pri2 | 0.806 |
| Pri3 | 0.804 |
| Pri4 | 0.807 |
| Enj1 | 0.811 |
| Enj2 | 0.811 |
| Enj3 | 0.805 |
| Enj4 | 0.806 |
| Ang1 | 0.774 |
| Ang2 | 0.771 |
| Ang3 | 0.773 |
| Ang4 | 0.774 |
| Anx1 | 0.78 |
| Anx2 | 0.773 |
| Anx3 | 0.77 |
| Anx4 | 0.777 |
| Hop1 | 0.776 |
| Hop2 | 0.775 |
| Hop3 | 0.774 |
| Hop4 | 0.775 |
| Bor1 | 0.776 |
| Bor2 | 0.776 |
| Bor3 | 0.774 |
| Bor4 | 0.778 |
